# Challenges in Reducing Bias Using Post-Processing Fairness for Breast Cancer Stage Classification with Deep Learning

**DOI:** 10.3390/a17040141

**Published:** 2024-03-28

**Authors:** Armin Soltan, Peter Washington

**Affiliations:** Hawaii Health Digital Lab, Information and Computer Science, University of Hawaii at Manoa, Honolulu, HI 96822, USA

**Keywords:** algorithmic fairness, post-processing method, equalized odds, equalized opportunity, deep learning, breast cancer

## Abstract

Breast cancer is the most common cancer affecting women globally. Despite the significant impact of deep learning models on breast cancer diagnosis and treatment, achieving fairness or equitable outcomes across diverse populations remains a challenge when some demographic groups are underrepresented in the training data. We quantified the bias of models trained to predict breast cancer stage from a dataset consisting of 1000 biopsies from 842 patients provided by AIM-Ahead (Artificial Intelligence/Machine Learning Consortium to Advance Health Equity and Researcher Diversity). Notably, the majority of data (over 70%) were from White patients. We found that prior to post-processing adjustments, all deep learning models we trained consistently performed better for White patients than for non-White patients. After model calibration, we observed mixed results, with only some models demonstrating improved performance. This work provides a case study of bias in breast cancer medical imaging models and highlights the challenges in using post-processing to attempt to achieve fairness.

## 1. Introduction

Cancer is the second leading cause of mortality worldwide. Breast cancer, lung cancer, and colorectal cancer account for 51% of all new diagnoses among women. Breast cancer has the highest death rate at 32%. However, this death rate is not consistent across different demographic groups. For example, the death rate for Black women is 41% higher than for White women [1].

Recent advancements in deep learning have led to the use of deep neural networks, such as convolutional neural networks (CNNs), for breast cancer prediction. This field is relatively vast, with several models developed to classify benign and malignant tumors as well as to classify the stage of cancer [2,3].

Unfortunately, the use of artificial intelligence (AI) for cancer diagnostics may increase health disparities [4]. Because AI models are trained using differing amounts of data for each demographic group, they have the potential to lead to unfair predictions for under-represented groups [5–11].

Three broad classes of algorithms have been investigated to mitigate bias in algorithmic fairness: pre-processing, in-processing, and post-processing. Pre-processing involves changing the data, such as by generative data augmentation, to create equal amounts of data for each demographic group prior to training the model [12,13]. In-processing methods change the learning algorithm’s optimization objective function to enforce a reduction in bias during the training process. These two categories of techniques can function well if modifications to the underlying data or training process are allowed [13,14].

The final category of methods, post-processing, is applied after the model has been trained, using a separate set of data that was not used during the training phase. Such “black box” approaches are ideal when determining whether modifying the original AI model is impossible or infeasible [13]. In this work, we explore the utility of applying post-processing fairness adjustments to breast cancer stage classification using medical imaging data, testing whether standard post-processing methods adapted to the multi-class setting can mitigate bias in these models.

We structure the remainder of the paper as follows: Section 2 provides a description of the AIM-Ahead dataset we used, the fairness metrics we measured, and the deep learning models we trained. Section 3 reports the results of our analyses, characterizing biases that occur across demographic groups and describing the results of post-processing fairness modifications. Section 4 discusses the high-level implications of this work.

## 2. Materials and Methods

### 2.1. Dataset

We used a dataset from AIM-Ahead containing whole slide images from 1000 breast biopsies from 842 patients from 2014 to 2020 [15]. Each unique dataset element is related to an individual biopsy.

These high-resolution images, with dimensions of 100,000 × 150,0000 pixels, are stored as NDPI files, averaging about 2 GB each. We used 10,856 whole slide images generated by 1000 biopsies, averaging five images per biopsy. Each slide is labeled according by the cancer stage associated with the biopsy. A total of 94% of these determinations were developed within one month of the biopsy procedure [15].

We randomly divided patients into two groups, with 80% of the data used for training and the remaining 20% reserved for evaluation. The dataset composition for binary classification is depicted in Table 1. The sub-dataset that is used for training consists of 328 biopsies collected from 234 patients, containing a total of 3273 slide images. The held-out dataset includes 41 biopsies from 41 patients and 367 slide images. We assigned a label of 1 to patients who have cancer stages 3 and 4 and a label of 0 to patients who do not show any symptoms of cancer.

Table 2 provides a breakdown of the training and held-out test sets when splitting the data according to a multi-stage classification formulation. In this case, we assigned a label of 0 to patients with stage 0 cancer, a label of 1 to patients with stage 1 or 2 cancer, and a label of 2 to patients with stage 3 or 4 cancer.

### 2.2. Machine Learning Models

**Figure 1.**
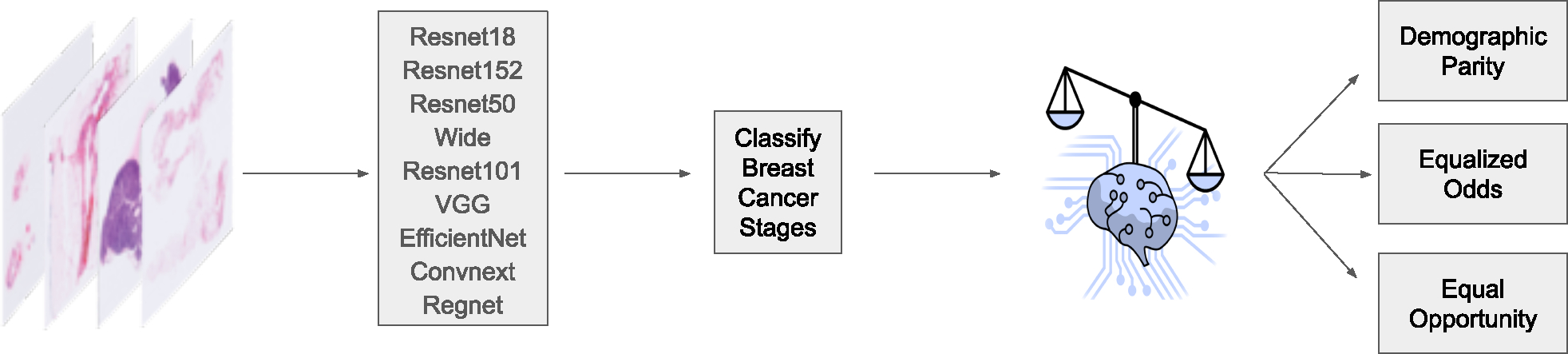
We used CNN models for image feature extraction and classification. We then applied post-processing strategies in an attempt to reduce bias. Finally, we evaluated the models using traditional algorithmic fairness metrics.

We evaluate a large number of important CNN architectures (Figure 1) for the classification of breast cancer stages from histopathological images. These architectures include VGG, EfficientNet, ConvNeXt, RegNet, and variations of ResNet models, including ResNet18, ResNet50, Wide ResNet101, and ResNet152. VGG stands out for its depth and use of numerous small-receptive-field filters that capture fine details. EfficientNet scales CNNs using a compound coefficient for balanced efficiency. ConvNeXt adapts Transformer principles for convolutional architectures, often enhancing performance. RegNet optimizes network structures for favorable performance/complexity ratios.

While we explored the possibility of training more modern model architectures, particularly Vit and Swin-Vit, on this dataset, our early attempts did not yield satisfactory results. This is likely due to the inadequacy of samples present in the dataset, which renders highly parameterized models ineffective, as highlighted by Zhu et al. [16]. We therefore did not pursue such architectures in our analysis.

**Figure 2.**
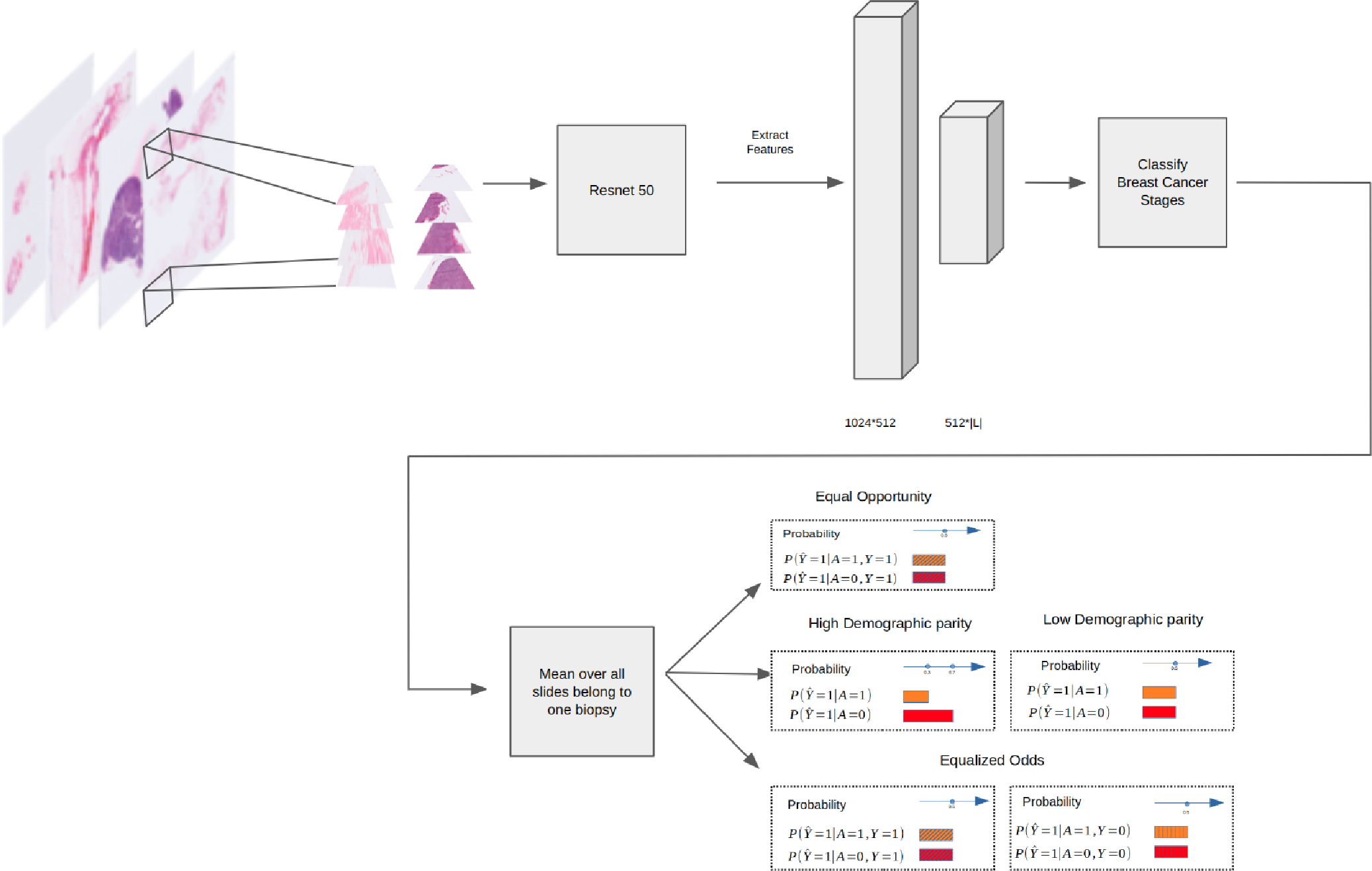
The workflow and architecture of our Slide Level Classifier: feature extraction, classification, and fairness-centered post-processing.

Our Slide Level Classifier, depicted in Figure 2, is tailored specifically to biomedical image data. We used Clustering-constrained Attention Multiple Instance Learning (CLAM). This weakly supervised method employs attention-based learning to automatically identify sub-regions of high diagnostic value to classify the whole slide. CLAM uses instance-level clustering over the representative regions identified to constrain and refine the feature space [17]. After retrieving features, we added two fully connected layers, with the first layer mapping the feature inputs to a 512-node hidden layer with ReLU activation. The second layer transforms the representation to the number of target classes. The classifier is further enhanced with feature pooling methods—average and max pooling—to synthesize information from the tile-level data of the slide images into a cohesive feature vector, which is then used for classification.

We also construct an Ensemble model, integrating the averaged predictions from all other models to produce a final outcome.

### 2.3. Fairness Definitions

Fairness metrics are crucial tools for evaluating and ensuring unbiased mitigation across all demographic groups, irrespective of race, gender, or other protected characteristics. We describe two common fairness metrics that we used to evaluate the bias of our models.

#### 2.3.1. Equalized Odds

Equalized odds is a fairness measurement for predictive models, ensuring that a predictor $\overset{ˆ}{Y}$ is independent of any protected attribute A given the true outcome Y. The measurement requires equal true positive and false positive rates across demographics in binary and multi-class settings. The purpose of equalized odds is to ensure that no group is unfairly advantaged or disadvantaged by the predictions.

##### Definition 1.

For binary variables, equalized odds is defined as:

(1) $$Pr(\overset{ˆ}{Y}=1|A=0,Y=y)=Pr(\overset{ˆ}{Y}=1|A=1,Y=y),\;y\in \{0,1\}$$

This metric aligns with the goal of training classifiers that perform equitably across all demographics [18].

#### 2.3.2. Equal Opportunity

In binary classification, Y=1 often represents a positive outcome, like loan repayment, college admission, or promotion. Equal opportunity is a criterion derived from equalized odds, focusing only on the advantaged group. It requires non-discrimination within this group, ensuring that those who achieve the positive outcome Y=1 have an equal probability of doing so, regardless of the protected attribute A. This is less stringent than equalized odds and often leads to better utility.

##### Definition 2.

*Equal opportunity for a binary predictor* $\overset{ˆ}{Y}$ *is defined as*

(2) $$Pr(\overset{ˆ}{Y}=1|{|}_{Y}=0,\Upsilon =1)=Pr(\overset{ˆ}{Y}=1|A=1,Y=1)$$

This condition mandates an equal True Positive Rate (TPR) for different demographic groups without imposing requirements on the False Positive Rate (FPR), thus allowing for potentially greater overall utility of the predictor [18].

We define FPR and TPR as follows, using TP to denote a True Positive, FP to denote a False Positive, TN to denote a True Negative, and FN to denote a False Negative:

(3) $$FPR=\frac{FP}{FP\;+\;TN}$$

(4) $$TPR=\frac{TP}{TP\;+\;FN}$$

### 2.4. Fairness Adjustments

We build our fairness adjustment method upon previous post-processing algorithmic fairness work. Hardt et al. [18] propose a method that helps to adjust the model’s outputs to ensure fairness when there are only two possible outcomes. Putzel et al. [19] suggest a way to adapt this method for situations with more than two outcomes, such as the breast cancer stage classification task that we study here. To mitigate the issue of sparse samples for some groups, as is the case with our dataset, we introduce a minor adjustment, an epsilon term, to the TPR and FPR calculations to avoid division errors. By analyzing predicted and true labels alongside sensitive attributes such as race, we engineer ‘adjusted’ predictions that meet predefined fairness criteria. The resulting predictors aim to balance false positive and true positive rates (for equalized odds) or synchronize true positive rates (for equal opportunity) to ensure fairness across different demographics.

We leverage ROC curves to discern optimal fairness thresholds. Aligning ROC curves across groups leads to predictors that fulfill equalized odds, whereas mismatches may necessitate varying thresholds or probabilistic adjustments to achieve fair treatment. We identify optimal predictors by analyzing the intersections of group-specific convex hulls formed from these ROC curves. We manipulate conditional probabilities within the protected attribute conditional probability matrices through linear programming, optimizing against a fairness-oriented loss function. This process also incorporates an element of flexibility, allowing the loss function to penalize inaccuracies differently based on protected group membership.

Our fair predictors ensure a balanced representation of demographic groups by equalizing various fairness metrics. We explore two different multi-class fairness criteria, although the method could generalize to other fairness metrics as well.

We aim to minimize the same expected loss function for multiple classification that was used by Putzel et al. [19]:

(5) $$E\left[l\left({\hat{y}}^{adj},y\right)\right]=\sum_{\alpha \in 𝒜}\sum_{i=1}^{\left|𝒞\right|}\sum_{j\neq i}W_{ij}^{\alpha }\mathrm{Pr}(A=\alpha ,Y=j)l(i,j,\alpha )$$

where $W_{ij}^{\alpha }=Pr(Y_{adj}=i|\overset{ˆ}{Y}=j,A=\alpha )$ are the protected attribute conditional confusion matrices.

To preserve fairness at the individual prediction level, we adopt a stochastic approach. Instead of simply selecting the most probable class, we construct predictions by sampling from the adjusted probabilities. Due to insufficient sample sizes within each demographic group, we encountered instances of zero values for FPs, TPs, FNs, and TNs. To implement our method, we used existing software for calculating fairness metrics, which was originally developed based on binary classification [13,20]. We add an epsilon term (0.001) to the denominator of each of the four measurements (FPs, TPs, FNs, and TNs) to prevent division errors when calculating the confusion matrix and the fairness metrics (equalized odds and equal opportunity).

### 2.5. Evaluation Procedure

To ensure statistical robustness, we employ 50 iterations of a bootstrapping approach. During each iteration, we randomly select a subset comprising half of the test samples. This subset is used to compute the FPR and TPR for White and non-White patient groups across all models.

We determine the mean, standard deviation, and confidence intervals of these metrics, allowing for a comparative analysis between the White and non-White cohorts. We apply the *t*-test to measure the statistical significance of the observed differences across groups.

## 3. Results

**Figure 3.**
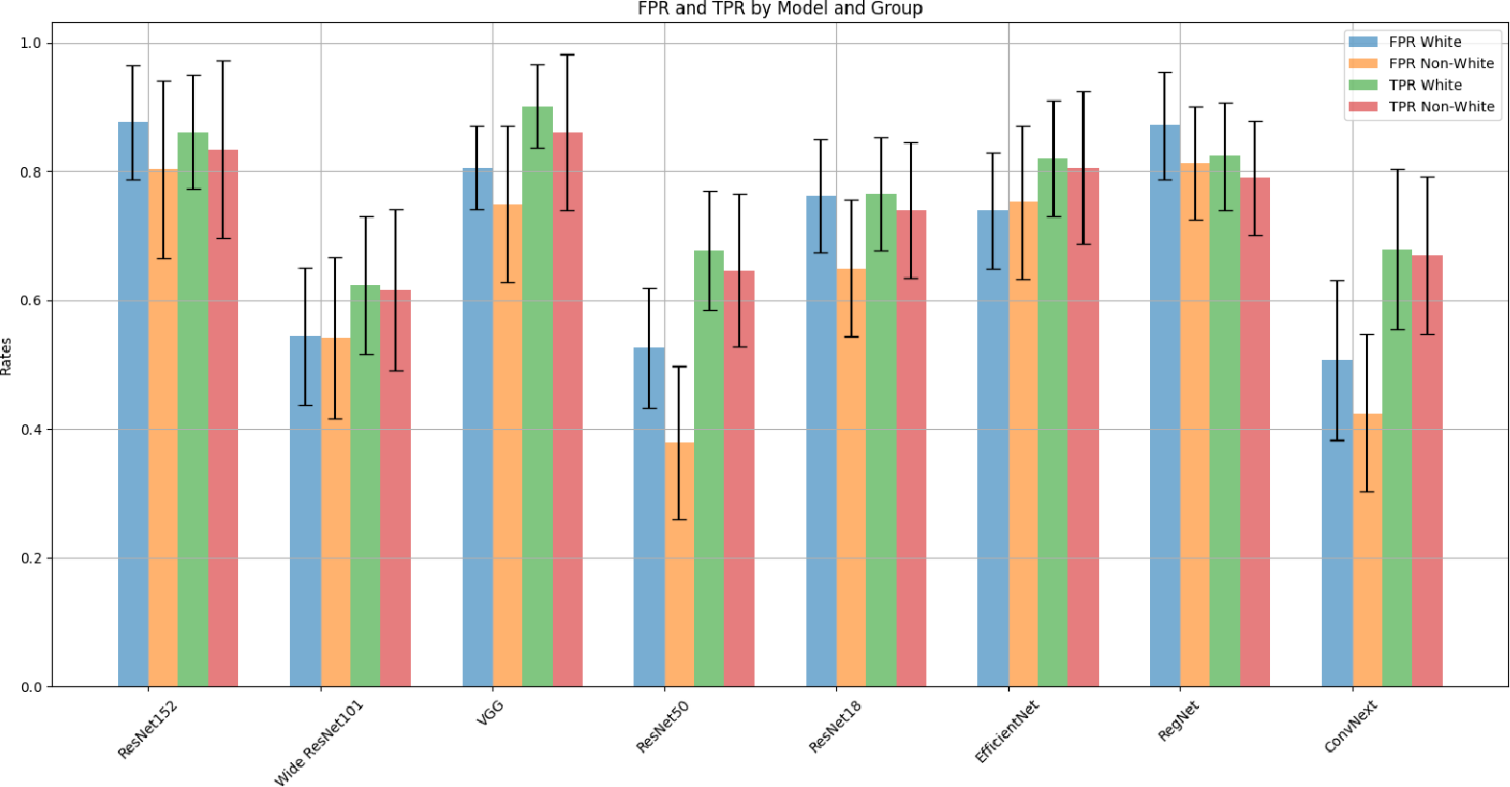
FPR and TPR for several binary deep learning models, distinguishing between White and non-White group performance.

Table 3 presents a comparative analysis, prior to fairness adjustments, of several binary classification deep learning models based on their performance metrics across two demographic stratifications of the dataset: White and non-White groups. We observe a consistent trend of higher binary accuracy, precision, and recall for the White group across all models. The Ensemble model achieves relatively high precision and recall for the White group but exhibits a significant drop in performance for the non-White group, especially in terms of accuracy and F1-score. These findings highlight the disparities in model performance for under-represented demographic groups and emphasize the need for more balanced and fair machine learning algorithms. Figure 3 illustrates this performance disparity in FPRs and TPRs among the various CNN models between groups.

Table 4 presents the results of independent *t*-tests conducted to compare the FPR and TPR between groups across various models before applying post-processing adjustment. The majority of the models show a statistically significant difference in FPR, highlighting concerns regarding biases in model performances across demographic groups. Although we did not consistently observe statistical significance at the 0.05 *p*-value cutoff for TPR, we note that the trend was always towards better performance for White groups, and some models still showed statistically significant differences in TPR. There were no models where the trend was reversed: that is, no models where the performance was better for the non-White groups.

**Figure 4.**
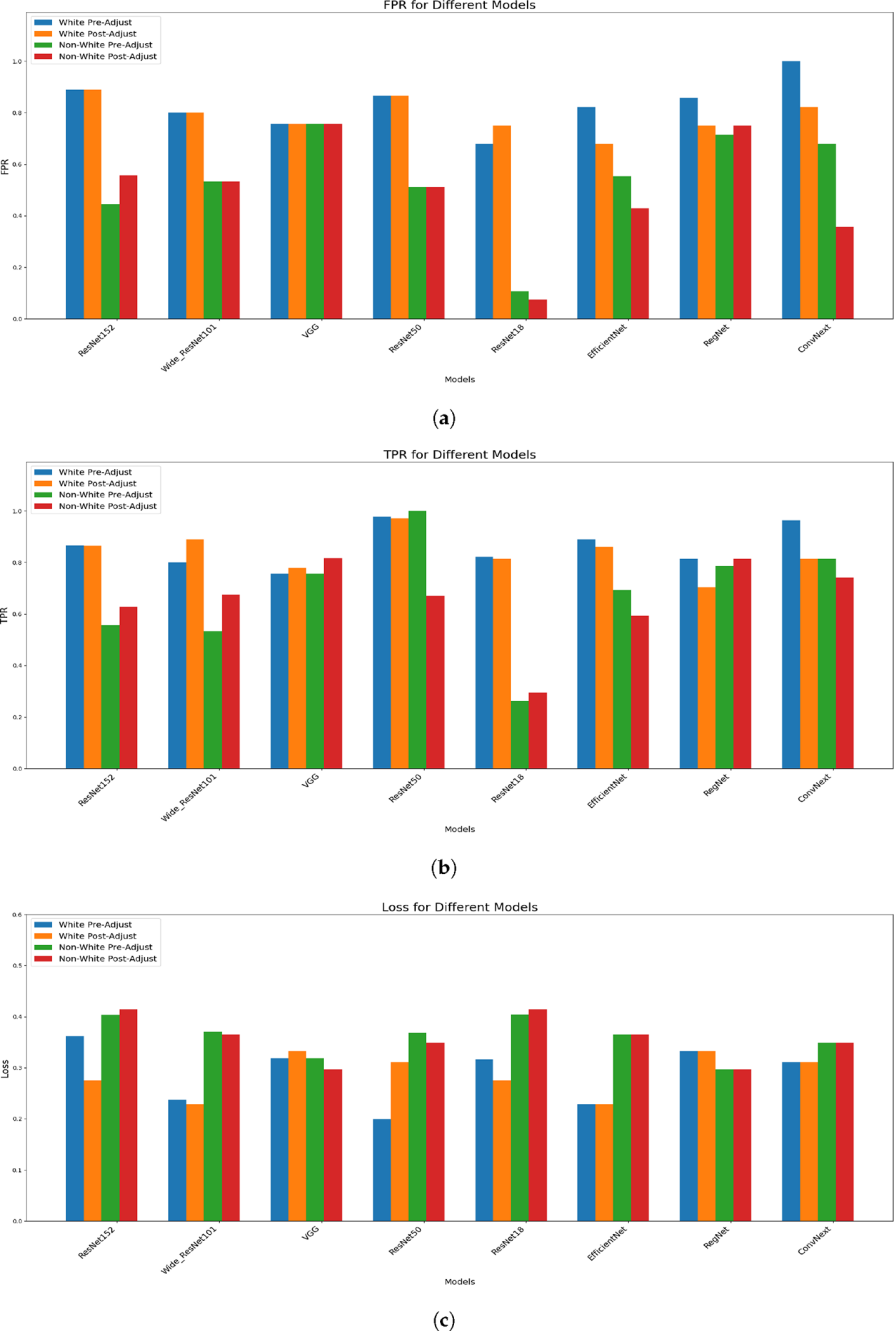
Comparative analyses across eight machine learning models, demonstrating the impact of fairness adjustments on the FPR, TPR, and loss. (**a**) FPR comparisons, (**b**) TPR comparisons, (**c**) loss comparisons. We do not observe consistent trends.

Table 5 and Figure 4 offer a comprehensive view of model performance before and after fairness adjustments in the binary classification setting. Notably, we do not observe consistent improvements in either FPR or TPR post-adjustment.

Table 6 provides an updated comparison of performance metrics for several models for the multi-class setting. The analysis was conducted across White and non-White groups for three different labels. We did not observe consistent discrepancies in performance between the White and non-White groups in the multi-class formulation, but we are hesitant to draw any conclusions from this due to the low overall performance of the models in the multi-class setting.

**Figure 5.**
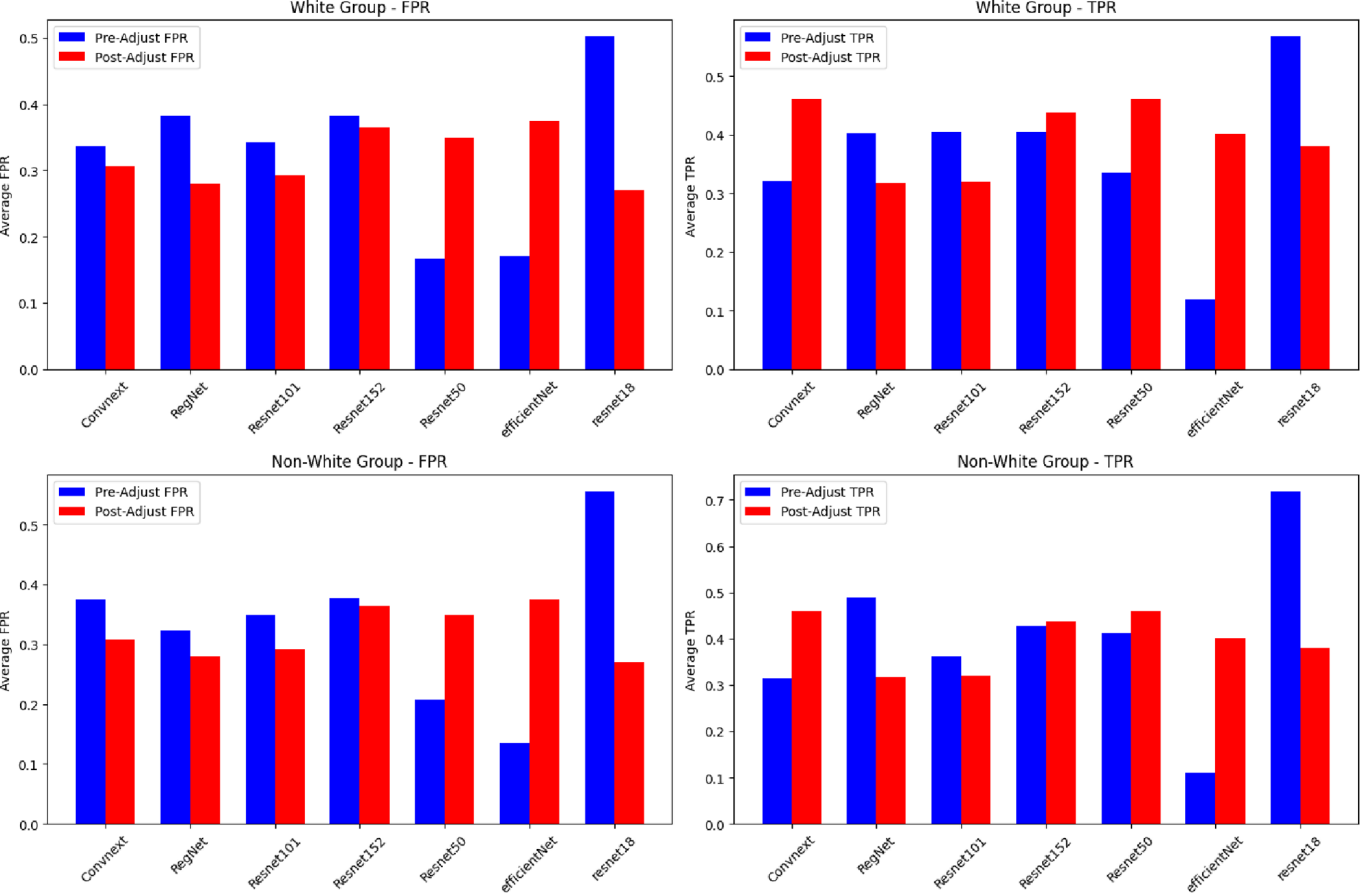
Comparative analysis of multi-class model performance across several architectures.

Figure 5 and Table 7 present a comparative analysis of the performance metrics for the deep learning models before and after fairness adjustments in the multi-class setting. We once again do not observe any improvements in performance after the fairness adjustments, but we are hesitant to draw any conclusions from the multi-class results due to the low overall baseline performance.

## 4. Discussion

We observe biases in the performance of the binary classification model, which consistently performs better on test data corresponding to White individuals. Our work adds further evidence to a wide body of prior work [21–23], demonstrating that without care, the integration of AI into diagnostic workflows may amplify existing healthcare disparities.

The lack of consistent disparity reductions after fairness adjustments highlights the challenges in applying post-processing techniques to reduce bias in machine learning models trained using medical imaging data. By adjusting the models after training, we had hoped to improve the equity of AI-enabled diagnostics across different racial groups. However, these methods do not appear to work for deep learning models applied to medical imaging.

The primary limitations of this study are (1) the small size of our evaluation dataset and (2) the possible lack of generalizability of our findings due to the use of only one dataset for evaluation. Future research on post-processing fairness in medical imaging would benefit from the use of multi-site datasets for a larger number of patients covering a broader range of demographic attributes. Another major limitation is that we grouped all non-White patients into a single category for fairness analyses due to the lack of sufficient representation of any race other than White. A more robust analysis would have included performance metrics for each individual race. However, such an analysis requires more samples for the under-represented groups, posing a ‘chicken-and-egg problem’. These limitations collectively render our study as a preliminary analysis that should be followed up with more expansive experimentation.

Another interesting area of future work would be studying the explainability of the models in conjunction with fairness. Such a study could aid in the understanding of how different models arrive at their predictions and whether the reasons for arriving at a particular prediction are different across groups.

## Figures and Tables

**Table 1.** Data distribution of training, validation, and test sets for the binary classification of no cancer from advanced-stage cancer.

	Train	Validation	Test

Biopsies	328	41	41
Images	3273	342	367
White	234	28	32
non-White	94	13	9
Biopsies with stage			
0	41%	49%	61%
III, IV	59%	51%	39%

**Table 2.** Data distribution of training, validation, and test sets for the multi-class classification formulation.

	Train	Validation	Test

Biopsies	800	100	100
Images	8847	967	1032
White	483	66	61
non-White	317	34	39
Biopsies with stage			
0	18%	16%	18%
I, II	58%	60%	66%
III, IV	23%	24%	16%

**Table 3.** Comparison of performance metrics across models for White and non-White groups prior to fairness adjustments.

Models	Groups	Accuracy	Precision	Recall	F1-Score

ResNet18	White	70.62 ± 2.83	0.76 ± 0.03	0.71 ± 0.03	0.73 ± 0.03
	non-White	55.27 ± 6.33	0.66 ± 0.10	0.55 ± 0.06	0.46 ± 0.08
ResNet50	White	67.37 ± 1.75	0.77 ± 0.02	0.67 ± 0.02	0.71 ± 0.02
	non-White	61.95 ± 4.31	0.74 ± 0.04	0.62 ± 0.04	0.57 ± 0.05
Wide ResNet101	White	58.31 ± 1.66	0.76 ± 0.02	0.58 ± 0.02	0.64 ± 0.02
	non-White	56.72 ± 4.24	0.57 ± 0.04	0.57 ± 0.04	0.57 ± 0.04
ResNet152	White	62.21 ± 1.46	0.75 ± 0.01	0.62 ± 0.01	0.67 ± 0.01
	non-White	55.96 ± 3.59	0.57 ± 0.04	0.56 ± 0.04	0.55 ± 0.04
VGG	White	65.84 ± 1.34	0.76 ± 0.01	0.66 ± 0.01	0.70 ± 0.01
	non-White	57.16 ± 2.55	0.59 ± 0.03	0.57 ± 0.03	0.56 ± 0.03
EfficientNet	White	67.34 ± 1.13	0.76 ± 0.01	0.67 ± 0.01	0.71 ± 0.01
	non-White	56.38 ± 3.23	0.58 ± 0.03	0.56 ± 0.03	0.55 ± 0.03
ConvNeXt	White	66.91 ± 0.84	0.77 ± 0.01	0.67 ± 0.01	0.71 ± 0.01
	non-White	57.29 ± 1.94	0.60 ± 0.02	0.57 ± 0.02	0.55 ± 0.02
RegNet	White	67.58 ± 0.97	0.76 ± 0.01	0.68 ± 0.01	0.71 ± 0.01
	non-White	56.09 ± 1.96	0.59 ± 0.02	0.56 ± 0.02	0.53 ± 0.02
Ensemble	White	62.88 ± 8.76	0.67 ± 0.09	0.63 ± 0.09	0.62 ± 0.09
	non-White	29.56 ± 13.26	0.79 ± 0.34	0.30 ± 0.13	0.34 ± 0.18
Slide Level	White	65.44 ± 8.18	0.68 ± 0.08	0.65 ± 0.08	0.65 ± 0.08
	non-White	55.11 ± 16.47	0.77 ± 0.24	0.55 ± 0.16	0.63 ± 0.17

**Table 4.** Results of independent t-tests comparing FPR and TPR between White (n = 32 in the test set) and non-White (n = 9 in the test set) groups across different models before applying post-processing adjustment.

Model	FPR		TPR	
	t-statistic	*p*-value	t-statistic	*p*-value
ResNet152	1.93	0.0606	1.93	0.0607
Wide ResNet101	0.06	0.9507	0.32	0.7474
VGG	1.87	0.0693	3.04	0.0043
ResNet50	3.93	0.0003	1.46	0.1514
ResNet18	3.24	0.0024	1.33	0.1923
EfficientNet	−0.35	0.7272	0.84	0.4034
RegNet	1.83	0.0746	1.87	0.0696
ConvNeXt	1.76	0.0860	0.47	0.6399

**Table 5.** Model performance before and after fairness adjustments in the binary classification setting. We compare the FPR, TPR, and loss values before adjustment and after applying two post-processing procedures optimized for equalized odds and equalized opportunity.

Model	Group	FPR			TPR			Loss		

		Pre-Adjust	Post-Adjust Odds	Post-Adjust Opportunity	Pre-Adjust	Post-Adjust Odds	Post-Adjust Opportunity	Pre-Adjust	Post-Adjust Odds	Post-Adjust Opportunity

ResNet152	White	0.8889	0.8889	0.8652	0.8667	0.8165	0.8652	0.2732	0.3161	0.2752
	non-White	0.6786	0.7500	0.7778	0.8214	0.8148	0.8889	0.2732	0.3161	0.2752
Wide_ResNet101	White	0.4444	0.5556	0.3745	0.5556	0.6367	0.6255	0.5683	0.4033	0.4142
	non-White	0.1071	0.5714	0.0000	0.5714	0.6296	0.5926	0.5683	0.4033	0.4142
VGG	White	0.8000	0.8000	0.8989	0.8000	0.8989	0.8989	0.2077	0.2371	0.2289
	non-White	0.8214	0.6786	1.0000	0.7500	0.8889	0.9259	0.2077	0.2371	0.2289
ResNet50	White	0.5333	0.5333	0.6742	0.5333	0.6742	0.6742	0.2842	0.3706	0.3651
	non-White	0.5357	0.4286	0.9630	0.5357	0.6296	0.5926	0.2842	0.3706	0.3651
ResNet18	White	0.7556	0.7556	0.7790	0.7556	0.7790	0.7790	0.2896	0.3188	0.3324
	non-White	0.8571	0.7500	0.9630	0.6786	0.8148	0.7037	0.2896	0.3188	0.3324
EfficientNet	White	0.7556	0.7556	0.8165	0.7556	0.7865	0.8165	0.2896	0.3188	0.2970
	non-White	0.7143	0.7500	0.7778	0.7143	0.7778	0.8148	0.2896	0.3188	0.2970
RegNet	White	0.8667	0.8667	0.824	0.9778	1.0000	0.8240	0.3333	0.3661	0.3106
	non-White	1.0000	0.8214	1.000	1.0000	0.9630	0.8148	0.3333	0.3661	0.3106
ConvNeXt	White	0.5111	0.5111	0.6704	0.5111	0.6704	0.6704	0.3497	0.3678	0.3488
	non-White	0.6786	0.3571	1.0000	0.5000	0.6296	0.7407	0.3497	0.3678	0.3488

**Table 6.** Comparison of performance metrics before post-processing adjustment for the multiple class formulation, stratified by race.

Models	Groups	Accuracy	Precision	Recall	F1-Score

ResNet18	White	37.50 ± 2.24	0.36 ± 0.03	0.38 ± 0.02	0.34 ± 0.03
	non-White	21.90 ± 1.78	0.73 ± 0.03	0.22 ± 0.02	0.24 ± 0.02
ResNet50	White	34.35 ± 1.83	0.40 ± 0.02	0.34 ± 0.02	0.36 ± 0.02
	non-White	36.64 ± 1.14	0.71 ± 0.02	0.37 ± 0.01	0.45 ± 0.01
Wide ResNet101	White	32.87 ± 1.26	0.39 ± 0.01	0.33 ± 0.01	0.35 ± 0.01
	non-White	42.13 ± 1.28	0.71 ± 0.01	0.42 ± 0.01	0.51 ± 0.01
ResNet152	White	37.70 ± 1.09	0.43 ± 0.01	0.38 ± 0.01	0.40 ± 0.01
	non-White	43.52 ± 0.94	0.71 ± 0.01	0.44 ± 0.01	0.52 ± 0.01
VGG	White	39.95 ± 0.89	0.43 ± 0.01	0.40 ± 0.01	0.41 ± 0.01
	non-White	37.51 ± 0.80	0.71 ± 0.01	0.38 ± 0.01	0.45 ± 0.01
EfficientNet	White	39.09 ± 1.13	0.42 ± 0.01	0.39 ± 0.01	0.40 ± 0.01
	non-White	38.04 ± 0.74	0.70 ± 0.01	0.38 ± 0.01	0.46 ± 0.01
ConvNeXt	White	39.08 ± 0.85	0.43 ± 0.01	0.39 ± 0.01	0.41 ± 0.01
	non-White	38.73 ± 0.70	0.70 ± 0.01	0.39 ± 0.01	0.47 ± 0.01
RegNet	White	38.66 ± 0.71	0.43 ± 0.01	0.39 ± 0.01	0.40 ± 0.01
	non-White	35.72 ± 0.65	0.70 ± 0.01	0.36 ± 0.01	0.43 ± 0.01
Ensemble	White	56.72 ± 6.26	0.44 ± 0.08	0.57 ± 0.06	0.49 ± 0.07
	non-White	69.23 ± 6.41	0.53 ± 0.08	0.69 ± 0.06	0.60 ± 0.08

**Table 7.** Model performance before and after fairness adjustments in the multi-class classification setting.

Model	Race	Class	FPR Pre-Adjust	FPR Post-Adjust	TPR Pre-Adjust	TPR Post-Adjust

RegNet	non-White	0	0.3577	0.0759	0.9375	0.1989
		1	0.1034	0.6321	0.0459	0.7160
		2	0.5598	0.2060	0.5000	0.2891
	White	0	0.4375	0.0759	0.5000	0.1989
		1	0.0738	0.6321	0.0549	0.7160
		2	0.4019	0.2060	0.5639	0.2981
ResNet50	non-White	0	0.3462	0.3304	0.5625	0.4375
		1	0.5172	0.5402	0.5872	0.5489
		2	0.0769	0.1154	0.0238	0.1219
	White	0	0.3304	0.3304	0.4375	0.4375
		1	0.5772	0.5402	0.5824	0.5489
		2	0.0748	0.1154	0.0902	0.1219
ConvNeXt	non-White	0	0.2692	0.5265	0.8125	0.8034
		1	0.3966	0.4441	0.3899	0.4262
		2	0.2735	0.0312	0.5000	0.0068
	White	0	0.2098	0.5265	0.5625	0.8034
		1	0.4228	0.4441	0.4066	0.4262
		2	0.2243	0.0312	0.4511	0.0068
ResNet152	non-White	0	0.0500	0.4895	0.0000	0.7500
		1	0.5000	0.4986	0.5046	0.4946
		2	0.4402	0.0000	0.5000	0.0000
	White	0	0.0357	0.4895	0.0000	0.7500
		1	0.5101	0.4986	0.5275	0.4946
		2	0.3832	0.0000	0.5038	0.0000
ResNet101	non-White	0	0.2231	0.4420	0.1250	0.5005
		1	0.6552	0.2012	0.5596	0.2422
		2	0.2137	0.3196	0.1429	0.3292
	White	0	0.2545	0.4420	0.1250	0.5005
		1	0.4832	0.2012	0.4176	0.2422
		2	0.2897	0.3196	0.3008	0.3292
EfficientNet	non-White	0	0.2462	0.3849	0.6875	0.6581
		1	0.5172	0.2245	0.4404	0.3421
		2	0.2821	0.2646	0.2143	0.3787
	White	0	0.2009	0.3849	0.5000	0.6581
		1	0.5705	0.2245	0.3956	0.3421
		2	0.3084	0.2646	0.2481	0.3787
ResNet18	non-White	0	0.0885	0.3341	0.0000	0.4074
		1	0.1034	0.5663	0.1835	0.5816
		2	0.7436	0.0812	0.7857	0.0952
	White	0	0.0670	0.3341	0.1250	0.4074
		1	0.1812	0.5663	0.1429	0.5816
		2	0.8131	0.0812	0.7218	0.0952

## Data Availability

The dataset used in this study is the publicly available dataset Nightingale Open Science Dataset, which can be found at the following website: https://app.nightingalescience.org/contests/8lo46ovm2g1j#dataset, accessed on 7 January 2024. All code is publicly available on Github (https://github.com/arminsoltan/AI_fairness_multiple_classification/tree/main (accessed on 27 March 2024)).
